# Uncovering complexity details in actigraphy patterns to differentiate the depressed from the non-depressed

**DOI:** 10.1038/s41598-021-92890-w

**Published:** 2021-06-29

**Authors:** Sandip Varkey George, Yoram K Kunkels, Sanne Booij, Marieke Wichers

**Affiliations:** 1 Department of Psychiatry, Interdisciplinary Center Psychopathology and Emotion regulation (ICPE), University of Groningen, University Medical Center Groningen (UMCG), Groningen , The Netherlands; 2 Faculty of Behavioral and Social Sciences, Department of Developmental Psychology, University of Groningen, Groningen, The Netherlands; 3 Center for Integrative Psychiatry, Lentis, Groningen, The Netherlands

**Keywords:** Psychology, Statistical physics, thermodynamics and nonlinear dynamics, Depression

## Abstract

While the negative association between physical activity and depression has been well established, it is unclear what precise characteristics of physical activity patterns explain this association. Complexity measures may identify previously unexplored aspects of objectively measured activity patterns, such as the extent to which individuals show repetitive periods of physical activity and the diversity in durations of such repetitive activity patterns. We compared the complexity levels of actigraphy data gathered over 4 weeks ($\sim 40000$ data points each) for every individual, from non-depressed ($n=25$) and depressed ($n=21$) groups using recurrence plots. Significantly lower levels of complexity were detected in the actigraphy data from the depressed group as compared to non-depressed controls, both in terms of lower mean durations of periods of recurrent physical activity and less diversity in the duration of these periods. Further, diagnosis of depression was not significantly associated with mean activity levels or measures of circadian rhythm stability, and predicted depression status better than these.

## Introduction

The association between physical activity and depression is well documented^1–3^. Group-level studies on levels of physical activity have shown an inverse association between physical activity and depressive symptoms^4–6^. Longitudinal studies, including intervention studies have shown that physical activity and exercise reduces symptoms and improves mood in individuals suffering from depression^7,8^. Studies have also shown that the association between depression and physical activity, may be bidirectional^8,9^. An unresolved question, however, is what precise characteristics of activity patterns are responsible for the impact of physical activity on mental health. Currently, most interventions work under the assumption that it is only the pure level of activity that impacts mood. Other studies, however, argue that, in addition to mean levels of physical activity, diurnal rhythms in activity are relevant in explaining why activity levels are associated with depression^10–12^. Healthy people are most active closer to the middle of the day and less active in mornings and evenings. Studies show that in depressed people, the activity rhythm peaks later than in healthy people^13,14^. The shifted timing influences sleep quality and diminishes levels of positive affect during the day^15,16^ and is hypothesized to contribute to depression. However, studies have been inconsistent and effect sizes are small^17,18^, suggesting that there may be other possible explanations for the association between activity patterns and depression. Currently, studies have only examined mean activity levels or activity rhythms with a constant periodicity (daily, weekly or annual activity rhythms) in association with depression^19–21^ and were not able to extract other sorts of recurring activity patterns, with varying periodicity, which may be relevant.

A new approach to examine activity patterns is to use tools from complexity science^22–24^. Complexity measures may provide new and complementary information on the nature of activity patterns in daily life as these measures are able to differentiate random activity spikes (noise) from patterns of activity that seem to repeat themselves, even if they vary in the timing of return (varying periodicity). Actigraphy patterns have been shown to be made up of a combination of intrinsic and extrinsic factors which give rise to activity time series that have a complex nonlinear mechanism overlaid with random fluctuations^25^. For example, restlessness behavior, which would generate movements at unpredictable moments is expected to show up as noise, rather than as specific repeating patterns of activity, whereas moments of sport, biking to work, or certain social activities that would involve elevated levels of physical activity would reveal themselves in repeating patterns of activity. Only these latter activity patterns add to the calculated complexity of the signal. Furthermore, not only the amount of repetition, but also the variety of repeating patterns adds to the calculated complexity of the measure. For example, differing durations of activities like biking, swimming or running would each cause a particular pattern of activity. When these activities are repeated in time, they constitute a diversity of recurrent physical activity patterns, which add to the complexity of the signal. Complexity measures would thus provide an objective way to measure to what extent these different types of physical activity (noise versus repeating activity patterns) are present in people with (risk for) depression. If we get a better understanding of what activity patterns differentiate depressed versus healthy people, this may not only provide more insight in how physical activity relates to depression, but, in the case that such patterns are causal to depression, it may also bring new possibilities for diagnostic tools to evaluate whether the patient exhibits healthy physical activity patterns. Moreover, complexity measures quantify an aspect of physical activity that is not captured by existing methods such as the mean activity levels or non-parametric circadian rhythm variables.

The study of physical activity patterns using small motion sensor detectors (accelerometers) that are encased in a unit about the size of a wristwatch and can be worn continuously for days to months, is called actigraphy^26^. Studying actigraphy patterns to understand mood disorders has become increasingly popular^27^. Actigraphs estimate levels of physical activity in an objective way, without recall bias^28,29^. Furthermore, the measurement of activity patterns using these light-weight devices, is non-invasive, with low burden to the participants, and therefore allows for the possibility of long-term monitoring of physical activity patterns.

In the current study, we aim to understand the differences in the complexity of recurrent physical activity patterns of depressed and non-depressed individuals using actigraphy. We hypothesize decreased levels of complexity in depressed people versus non-depressed people, in line with the argumentation given above. This reduced complexity can be measured using a lower duration and diversity of recurrent activity patterns in the physical activity data. For this purpose, we will use a unique sample of depressed and non-depressed people who were monitored for a month with accelerometers.

## Methods

### Sample

The data used were collected as part of the Mood and Movement in Daily Life (MOOVD) study, which aims to study the dynamic association between mood and physical activity^30,31^. All participants were aged between 20 and 50, and were monitored for 30 days using electronic diaries, actigraphy and saliva samples. In this paper we will use the actigraphy data and the diagnostic interview data on depression diagnosis. Data were obtained from 54 participants (depressed to non-depressed ratio 1 : 1) who were pair matched on gender, BMI, smoking status, and age. The participants were screened for severity of depression based on their scores on the Beck Depression Inventory (BDI)-II questionnaire^32^. Scores below 14 are associated with minimal depression, while scores of 14 and above are associated with mild, moderate or severe levels of depressive symptoms. Participants scoring above 14 and participants scoring below 9, were invited for a diagnostic interview to establish whether they fulfilled the criteria for depression or were free of any mood disorders, respectively. For further details, we refer the reader to Booij et. al (2015)^30^.

The MOOVD study design was approved by the Medical Ethical Committee of the University Medical Center Groningen. All participants gave written informed consent. The study was conducted in accordance with the Declaration of Helsinki.

### Measurements

Physical activity was measured using the ActiCal (Respironics,Bend,OR) which is an omni-directional, water-resistant actigraph, which was worn on the non-dominant wrist. The activity counts were sampled at 1 min-intervals and were used as the measure for physical activity. Details of how activity measurements are conducted in ActiCal can be found in Heil, 2006^33^.

### Data pre-processing

Prior to the main analysis, we carried out two preprocessing steps. The first reduced the overall size of the data by resampling. This is achieved by averaging the data through 10-min bins. This averaging or binning step gave us the average activity counts every 10 min, which reduced the total length of the time series, and computational time needed by the algorithm. To maintain uniformity, all datasets were constrained to a length of 4000 data points after binning, which gave us nearly 28 days or 4 weeks of data per participant. All datasets were ensured to have at least 3000 data points or about 21 days of data. A second preprocessing step involved a rank transformation on the data^34–36^. This analysis focused on methods that depend mostly on the ordering and rhythms in the time series. Hence we rank transformed all the datasets initially, resulting in a uniform amplitude distribution. The resulting transformation preserved the rank and time ordering and consequently the dominant periodicities of the time series. Using the rank transformation made sure that the quantifiers derived from the activity counts time series are not affected by extreme events, such as sudden spurts in activity. In addition the transformation put all the time series from different subjects onto an equal footing when it came to amplitude. This became especially useful in the context of the choice of a recurrence threshold, which we describe in the next subsection. The results from this rank transformed data were then only related to the ordering of the time series and not to the actual amounts of activity counts by a subject. This conversion was done by replacing a point in the time series by its rank in the time series. The resulting time series of ranks was then divided by N, which was the total length of the time series, which constrained the distribution between 0 and 1.

### Recurrence quantification analysis

Our analysis is primarily focused on the recurrence quantification analysis (RQA) of actigraphy data. It quantifies the relative abundance, duration and diversity of recurrent patterns in a time series. This kind of analysis has proved to be very useful in many different fields in science, including psychology^37–39^.

We conducted an RQA on all individuals in our sample. Then we compared the recurrence plot properties of the non-depressed and depressed groups with regard to complexity measures. Recurrence plots are simple binary plots that visualize the pattern of repetitions or rhythms in a time series. Fourier transform based methods capture repetitions that are periodic, whereas circadian rhythm variables consider rhythms at a day level. RQA is free from these constrains and the exact patterns in a recurrence plot give us a deeper understanding about the nature of the underlying dynamics that the time series is derived from.

A recurrence plot reveals the patterns a system makes when it revisits the same neighborhood of space. When the dynamics of a system is purely stochastic, the recurrence plot shows no discernible patterns. On the other hand, when the system shows deterministic behavior the recurrence plot shows distinct patterns in the form of horizontal and diagonal lines. These are quantified using RQA.

**Figure 1:**
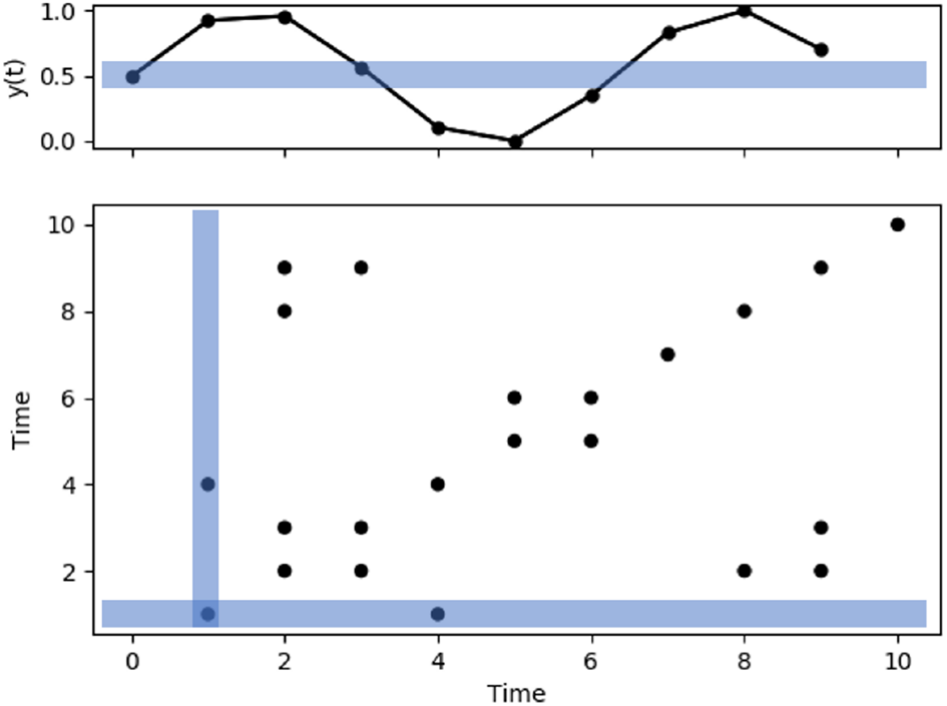
Schematic describing the construction of a recurrence plot. The upper panel shows the series of observations vs time. A region centered on the first data point, with width $2\epsilon$ is shaded in blue. The lower panel shows the corresponding recurrence plot. The elements of the recurrence plot corresponding to the first point are shaded in blue, in the lower panel. When a point falls within the blue rectangle in the upper panel, it is shown as a black point in the lower panel. This analysis is repeated for every point in the time series resulting in the complete recurrence plot. The x and y axes of the recurrence plot represent the time of observation (x axis of the upper panel). Hence, when an observation $y(t_1)$ at time $t_1$ and $y(t_2)$ at time $t_2$ are within $\epsilon$ distance of each other, the point $(t_1,t_2)$ is marked in black in the recurrence plot.

A recurrence plot is constructed in the following way. A recurrence threshold or distance is first chosen, say $\epsilon$. The time series to be analyzed, which in this study is the pre-processed actigraphy data, is then scanned such that all points that fall within $\epsilon$ distance of each time series point is identified. The recurrence plot is then generated as a planar plot of ordered time along the x and y axes. A schematic describing this process is shown in Fig. 1, where a region in blue, of size $2\epsilon$, is marked in the time series in the upper panel to demonstrate this recurrence threshold. If the amplitudes of two time series points fall within $\epsilon$ distance of each other, the corresponding point is marked using a dark spot in the recurrence plot. In the time series in Fig. 1, all points within the blue rectangle in the upper panel are marked as black points within the shaded region in the lower panel. The RQA in this paper is conducted using the free standalone software, TOCSY(Available from tocsy.pik-potsdam.de)^40,41^.

**Figure 2:**
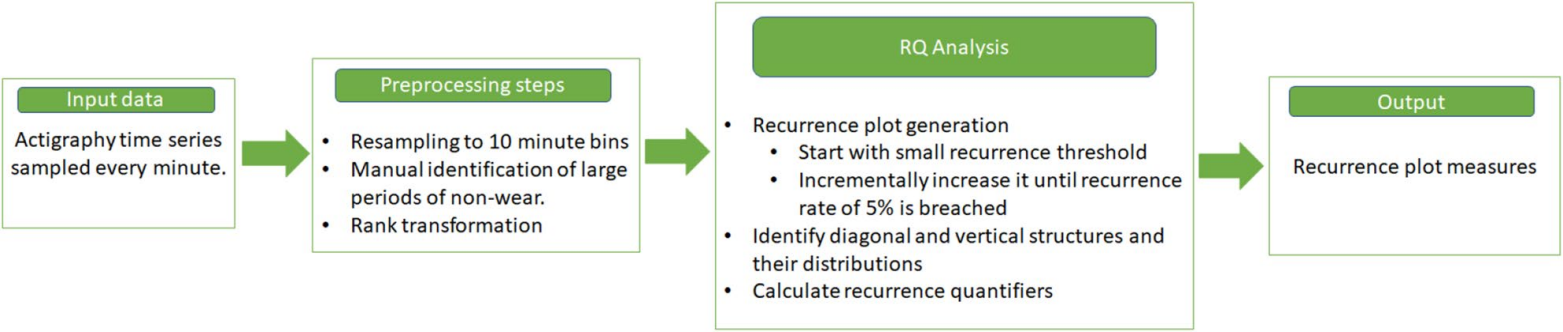
Flowchart indicating the analysis procedure, described in this paper, to extract recurrence plot measures from actigraphy data.

In this study, we set the recurrence threshold by constraining the recurrence rate or density of dark points in the plot. The recurrence rate gives a probability that a specific state will recur. For our study we set the recurrence threshold as the distance where the density is 0.05, i.e. about $5\%$ of the recurrence plot is made of dark points. A fixed density has been used previously in multiple studies to determine $\epsilon$ and is known to be useful in detecting finer changes in the recurrence plot structure^42–44^. A flowchart describing the process is shown in Fig. 2.

Once the recurrence threshold is fixed, we quantify two main structures in the recurrence plot, the diagonal and vertical lines. The diagonal line structures in the recurrence plot are associated with the level of determinism in the time series, since random processes will show these structures very rarely, whereas deterministic processes tend to show these structures more. It occurs either when a part of the time series changes monotonically or when two parts of the time series show similar local evolution or change. The vertical lines on the other hand indicate periods of “stasis” or very slow evolution. In a sense, it shows the length of the activity, with longer vertical lines suggesting an activity that lasts for longer. We are primarily interested in the mean and entropy of the distributions of the diagonal and vertical line lengths. The average of the diagonal line distribution shows the average duration of recurring physical activity patterns in a time series. The entropy quantifies the diversity associated with the diagonal structures in the recurrence plot. This provides a measure of the extend of time scales involved in the diagonal line distribution. Similarly the mean of the vertical line distribution shows the mean levels of stasis associated with the physical activity patterns (i.e how long an activity persists) and the entropy yields the diversity associated with the vertical line distribution^40^. Another important quantifier that is associated with the diagonal line structure is called the determinism or DET measure. The DET measure reflects the ratio of points that form diagonal structures to the ratio of all recurring points. Thereby, it provides an estimate of how often different parts of a time series co-evolve as a fraction of the total number of data point pairs in the plot. For a purely noisy process, with no underlying dynamics, this measure is very small, whereas for a process with underlying deterministic dynamics, the DET measure is high. Similarly, the laminarity or LAM measure reflects the ratio of points that form vertical structures to the ratio of all recurring points. This provides an estimate of how often slowly evolving processes occur, as a fraction of the total number of data point pairs. For instance, frequent periods of rest or physical activity that results in constant activity counts for an extended duration will lead to a higher LAM measure as opposed to cases when such patterns are rare. A further useful quantifier in this context is the ratio of the LAM to DET measure, which quantifies how often vertical structures appear in the system as a fraction of diagonal structures^45^. While all the quantifiers mentioned above relate to the complexity of patterns found in the recurrence plot, the mean and entropy of the distributions relate directly to the duration of recurrent activity patterns and the diversity of such patterns. A summary of the recurrence-based quantifiers used in this paper is given in Table 1.

**Figure 3:**
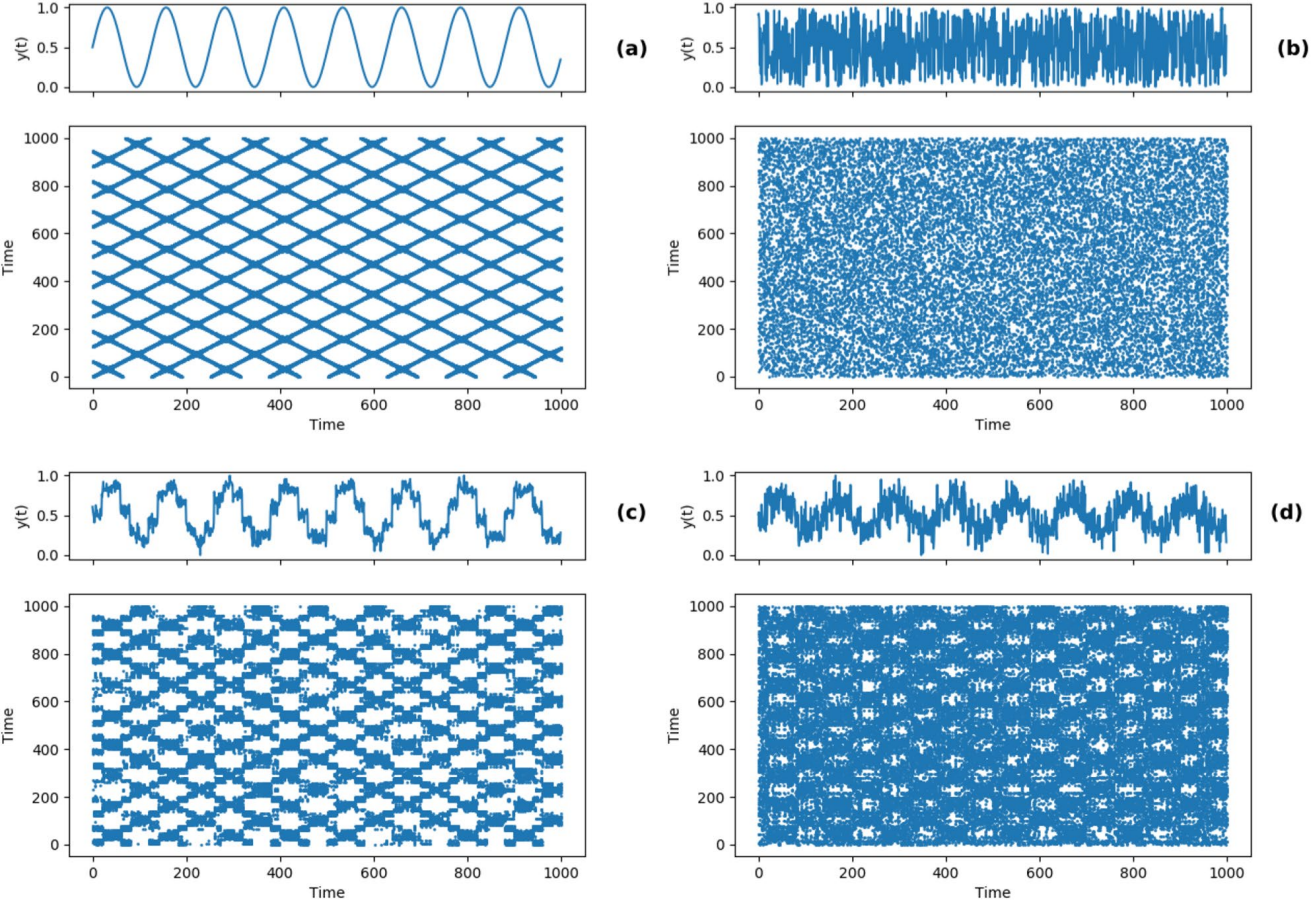
Sample time series and corresponding recurrence plots for (**a**) a pure sine wave (**b**) random noise (**c**) sine wave contaminated with additive white noise with signal to noise ratio (SNR) 5 and (**d**) sine wave contaminated with additive white noise with signal to noise ratio (SNR) 1. A higher SNR implies that the signal is more prominent as compared to the noise.

To illustrate the difference between time series data that is dominated by noise processes and one which is dominated by a periodic signal, we contaminated a sine wave by adding varying levels of white noise to them. The level of noise contamination is measured as the ratio of the mean squared expectation of the signal to noise (signal to noise ratio or SNR). Sample recurrence plots from these noise contaminated sine waves (SNR 5 and 1), along with a pure sine wave and a pure white noise signal, are shown in Fig. 3. The simulation of sine waves contaminated with noise shows instances where a strong rhythm along with randomness is present, similar to daily rhythms which are prominent in actigraphy data. As the random component becomes larger, the recurrence plot becomes more diffused.

**Table 1:** Definitions and interpretations in the context of activity data, for various recurrence plot quantifiers that are used in this work.

Quantifier	Calculation	Definition	Interpretation
*DET*	Ratio of diagonal structures to total recurrence points.	Level of deterministic activity in the data.	Lower levels indicate more randomness
*LAM*	Ratio of vertical structures to total recurrence points.	Level of slowly evolving processes in the time series.	Higher levels indicate more activities that linger
$L_{avg}$	Mean length of diagonal structures	Average duration of recurrent physical activity	Higher levels indicate longer recurrent physical activities
$L_{ent}$	Entropy of diagonal line distribution	Diversity of durations of recurrent physical activity patterns	Higher levels indicate recurrent physical activities of varying durations
$V_{avg}$	Mean length of vertical structures	Average duration of static activity patterns	Higher levels indicate lingering physical activities that last longer
$V_{ent}$	Entropy of vertical line distribution	Diversity of durations of static activity patterns	Higher levels indicate lingering physical activities of varying durations
$\frac{LAM}{DET}$	Ratio of LAM to DET measures	Level of statis as compared to deterministic structure	Changes in this ratio has been shown to be an indicator of change in stability^45,46^.

*DET*: Determinism; *LAM*: Laminarity; $L_{avg}$: Average diagonal line length; $L_{ent}$: Entropy of diagonal line distribution; $V_{avg}$: Average vertical line length; $V_{ent}$: Entropy of vertical line distribution; $\frac{LAM}{DET}$: Laminarity to determinism ratio.

### Missing data

Many datasets showed periods of non-wear in the beginning or the end of the collection period. Such periods were removed through visual inspection. Datasets that were left with less than 3000 measurement points, after resampling into 10-min bins, were eliminated initially. After the recurrence plot construction, all datasets for which the density threshold of 0.05 was exceeded at very low recurrence thresholds, were eliminated. This happens when the dataset has considerable periods of inactivity, which leads to cluttering in the recurrence plot.

### Traditional actigraphy quantifiers

In order to check for differences in discriminative ability and overlap between the current complexity measures and more traditional measures such as mean levels of activity and circadian rhythm variables, the latter variables were extracted as well from the actigraphy data. Both the mean and circadian rhythm variables have been used in differentiating healthy and depressed individuals from actigraphy data. The mean activity was calculated as the average number of activity counts per individual. The circadian rhythm variables used were the interdaily stability (IS), intradaily variability (IV) and relative amplitude (RA)^47^. The IS quantifies stability of the rhythm between days. It can vary between 0 and 1, with higher values indicating a more stable daily rhythm. The IV indicates the fragmentation of the sleep-wake rhythm and varies roughly between 0 and 2. Higher values indicates higher fragmentation. The RA gives a description of how different the most active and least active periods in a day are. Further details about calculation may be found in^48^. Circadian measures were calculated using the ACTman package in R version 3.6.3^49^.

### Statistical analysis

Group differences in complexity were examined using a t-test. The t-test for independent samples checks if two independent groups have identical mean values. We use the Welch t-test which does not assume equal population variance, and generalizes to unequal sample sizes^50^. The effect size was measured using the Cohen’s d^51^.

The independence between the different measures used in the study was calculated using the Spearman rank correlation coefficient. Apart from being robust to outliers, rank correlation coefficients have the added advantage that they find a correlation even if the monotonic relationship between the covariates is nonlinear^36^. The p-value for significance was set at 0.05. The t-tests and correlations were performed using the scipy package in python version 3.5.2^52^.

We also used logistic regression to predict the diagnostic status with traditional actigraphy quantifiers and recurrence quantifiers. The pseudo-$R^2$ values were then compared between the different quantifiers to quantify goodness of fit. The logistic regression was conducted using R version 3.6.3.

## Results

**Figure 4:**
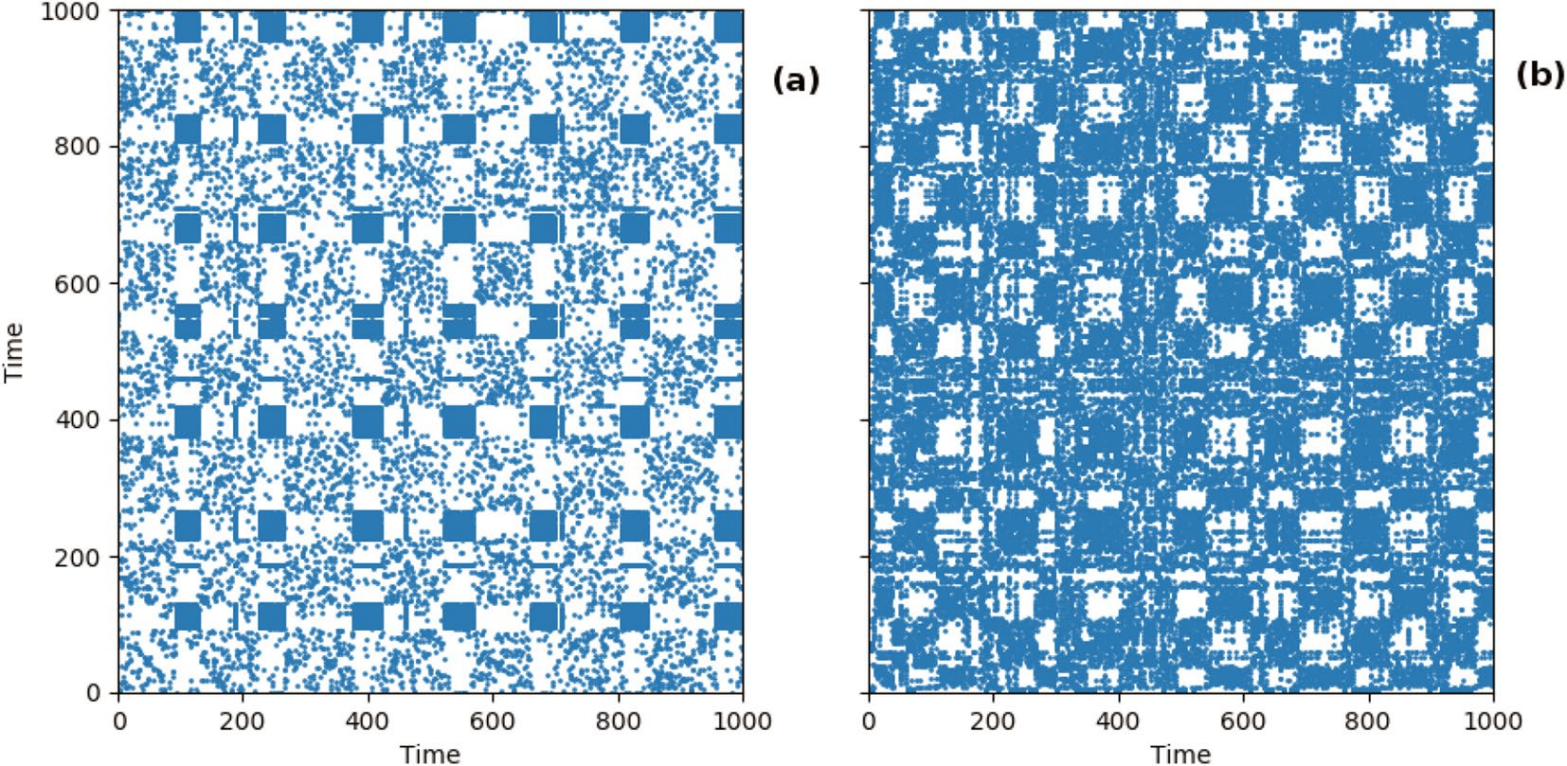
Sample recurrence plots for two individuals. (**a**) Shows the recurrence plot constructed from a non-depressed individual and (**b**) shows one constructed from a depressed individual.

We present the results of the between-group analysis comparing the RQA measures of the depressed group with the non-depressed group. One dataset was excluded initially due to insufficient data ($< 3000$ points). Another seven datasets were excluded due to cluttering in the recurrence plot which led to a recurrence rate larger than 0.05 even at very low values of the $\epsilon$ threshold. This left 21 depressed and 25 non-depressed participants. Sample recurrence plots from a non-depressed and a depressed individual are presented in Fig. 4.

### Descriptives and traditional actigraphy differences between the groups

Differences in demographic and clinical characteristics between the depressed and non-depressed subjects are shown in Table 2. In line with the fact that demographic variables are pair matched in the MOOVD study, we did not observe any significant differences. In Table 3 we showed the group differences between commonly used quantifiers of actigraphy, namely the mean activity counts and nonparametric circadian rhythm variables for actigraphy analysis proposed in^47^. No significant differences for these quantifiers between the two groups were observed, and all effect sizes were small to medium at best^51^.

**Table 2:** Differences in means for demographic and clinical measures for the non-depressed (n=25) ($Mean_N$) and depressed (n=21) groups ($Mean_D$).

Demographic and clinical characteristics	$Mean_N$ ± SD	$Mean_D$ ± SD	t-statistic	p-value
Age	$33.3\pm 8.9$	$33.2\pm 9.3$	$-0.030$	0.976
BMI	$22.33\pm 2.64$	$24.27\pm 6.31$	1.315	0.200
Gender	24% male	29% male	-0.384 (z-statistic)	0.351
Pre-BDI-II	$2.2\pm 13.2$	$29.3\pm 9.2$	13.045	<0.001
Post-BDI-II	$2.3\pm 3.4$	$20.4\pm 11.4$	6.987	<0.001

The pre and post BDI-II scores are the BDI-II scores before and after the data collection, respectively.

BDI-II: Beck Depression Inventory-II score, $Mean_N$: Mean of the non-depressed group, $Mean_D$: Mean of the depressed group, SD:Standard deviation.

**Table 3:** Differences in means of traditional quantifiers of the actigraphy time series for the non-depressed ($n=25$) and depressed ($n=21$) groups.

Quantifier	$Mean_N \pm SD$	$Mean_D \pm SD$	t-statistic	p-value	Cohen’s d
Average activity	262.388±77.570	228.040±84.319	− 1.427	0.161	0.435
IS	0.368±0.325	0.424±0.367	0.542	0.591	− 0.166
IV	1.414±0.65	1.256±0.55	− 0.891	0.378	0.265
RA	0.905±0.075	0.896±0.073	0.393	0.696	− 0.117

IS: Interdaily stability; IV: Intradaily variability; RA: Relative Amplitude; $Mean_N$: Mean of the non-depressed group; $Mean_D$: Mean of the depressed group; SD:standard deviation.

### Results of recurrence quantification analysis

We then checked the mean differences in the recurrence plot parameters for the two groups. A significant ($p < .05$) difference between the two groups in the mean and entropy of the diagonal line length distribution and in the ratio of LAM to DET was found. The depressed group showed lower mean and entropy for the diagonal line distribution, whereas it showed a larger LAM to DET ratio, compared to the non-depressed group.

**Figure 5:**
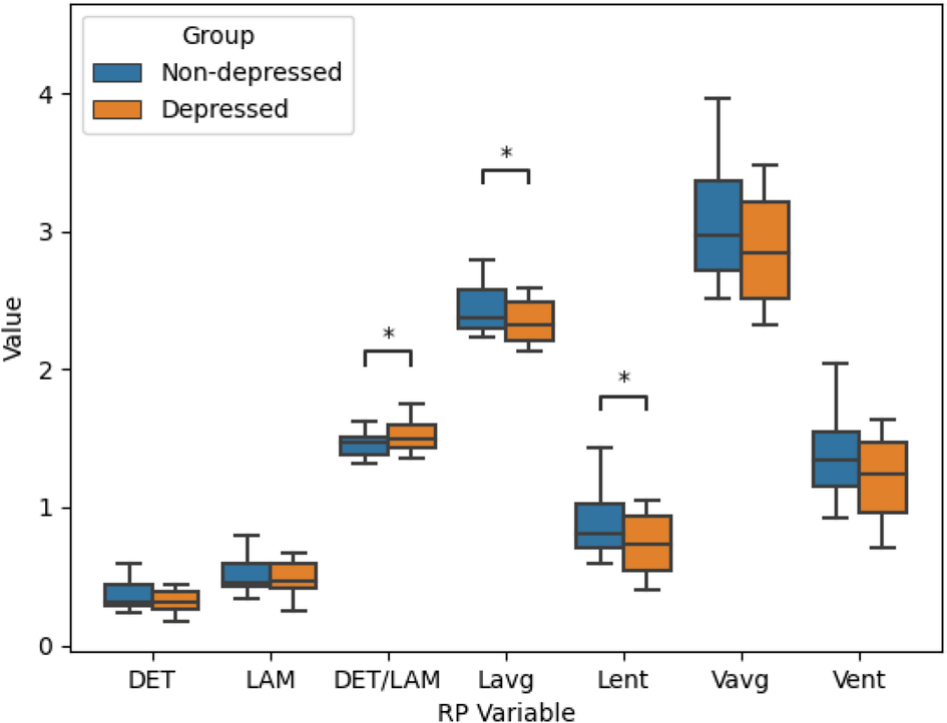
Box plots showing the differences between the non-depressed and depressed groups for the different recurrence plot variables. Significant differences ($p<.05$) are marked with asterisks ($*$).

**Figure 6:**
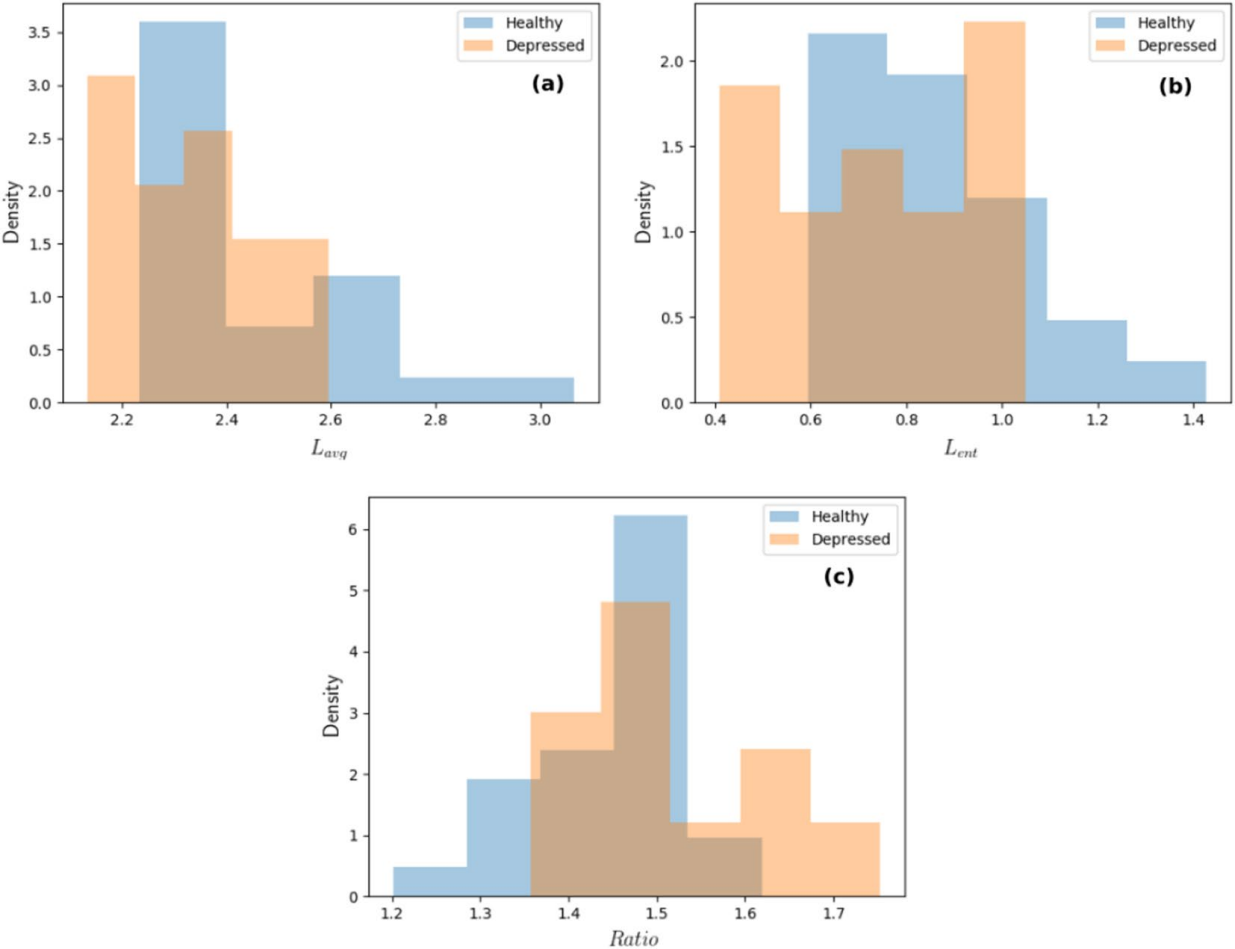
Histograms showing the difference in (**a**) mean diagonal length, (**b**) entropy and (**c**) ratio of determinism to laminarity between the non-depressed and depressed groups.

Overall, it was found that almost all the complexity measures were either significant ($p<.05$) or borderline significant ($p < .1$) and in the expected direction. Further all the quantifiers, except LAM, showed medium to large effect sizes^51^. The means of the considered measures for the two groups and the corresponding t-statistic, p-value and effect size are shown in Table 4 and the corresponding box-plots are shown in Fig. 5. The distributions of the three measures which showed significant differences between the depressed and non-depressed groups are shown in Fig. 6. A Bonferroni correction in the level of $\alpha$ for testing significance when conducting multiple tests causes all significant results to be lost.

**Table 4:** Differences in means for the different recurrence plot measures for the non-depressed($n=25$) and depressed($n=21$) groups.

Property	$Mean_N \pm SD$	$Mean_D \pm SD$	t-statistic	*p*-value	Cohen’s d
*DET*	0.359±0.11	0.308±0.087	− 1.725	0.091	0.511
$L_{avg}$	2.436±0.195	2.334±0.142	− 2.018	0.050*	0.594
$L_{ent}$	0.853±0.21	0.725±0.202	− 2.104	0.041*	0.634
*LAM*	0.511±0.13	0.466±0.119	− 1.168	0.249	0.353
$\frac{LAM}{DET}$	1.446±0.09	1.517±0.11	2.336	0.025*	− 0.721
$V_{avg}$	3.079±0.475	2.864±0.367	− 1.731	0.090	0.512
$V_{ent}$	1.352±0.28	1.206±0.257	− 1.707	0.095	0.520

The measures that show statistically significant ($p<.05$) differences are indicated with an *

*DET*: Determinism; $L_{avg}$: Average diagonal line length; $L_{ent}$: Entropy of diagonal line distribution; *LAM*: Laminarity; $\frac{LAM}{DET}$: Laminarity to determinism ratio; $V_{avg}$: Average vertical line length; $V_{ent}$: Entropy of vertical line distribution, $Mean_N$: Mean of the non-depressed group; $Mean_D$: Mean of the depressed group; SD: Standard deviation.

A logistic regression was used to check whether the individual recurrence measures predicted the depression status better than the traditional actigraphy measures. The recurrence measures were found to have higher pseudo $R^2$ values than traditional actigraphy measures. Moreover, using a combination of traditional actigraphy measures and novel recurrence plot based actigraphy measures yielded a higher $R^2$ value than each did individually, suggesting that they provide complementary information. For details, please refer to the supplementary material.

### Correlations

Finally, the correlations of the recurrence plot parameters with the traditional variables considered earlier in this section and with each other were analyzed to examine the extend of overlap between these quantifiers. These are listed in Table 5. We see a perfect correlation between the mean and entropy measures of the diagonal line distribution. Correlations between average level of physical activity and diurnal rhythm variables on the one hand and complexity measures on the other hand were generally small. One weak but significant correlation was found between the interdaily stability and the LAM to DET ratio.

**Table 5:** Correlation between commonly used actigraphy measures and recurrence plot measures .

	$L_{avg}$	$L_{ent}$	$\frac{LAM}{DET}$	Avg Activity	IS	IV	RA
$L_{avg}$	1	0.999*	− 0.609*	0.091	0.073	0.031	0.201
$L_{ent}$		1	− 0.607*	0.107	0.073	0.035	0.206
$\frac{LAM}{DET}$			1	0.286	− 0.314*	− 0.098	0.013
Avg Activity				1	− 0.162	− 0.132	0.157
IS					1	0.355*	− 0.140
IV						1	− 0.185
RA							1

Only the recurrence plot measures that showed significant differences between the two groups are listed.

The table lists the Spearman correlation coefficient. Significant correlations(*p* < 0.05) are marked with an *.

IS: Interdaily stability; IV: Intradaily variability; RA: Relative Amplitude; $L_{avg}$: Average diagonal line length; $L_{ent}$: Diagonal line entropy; $\frac{LAM}{DET}$: Laminarity to determinism ratio.

## Discussion

This work explored how complex recurrent patterns in physical activity are associated with depression. Significant differences between the non-depressed and depressed groups for multiple recurrence plot quantifiers, which were related to duration and diversity of physical activity patterns were observed. Using RQA an overall lower level of the complexity of recurrent longitudinal patterns in depressed patients versus controls was shown. While the study does not conclusively prove differences in complexity between the two groups, especially after taking into account multiple testing corrections, it does leave room for cautious optimism about using these quantifiers to study depression using physical activity data, in future research.

The current work represents an important first step in multiple ways. The methodology used in this work goes beyond the way how classical approaches related activity patterns to depression. By using novel methods from complexity science, we were now for the first time able to capture other relevant aspects of recurrent temporal patterns of physical activity such as the duration and diversity of such activity patterns that have varying periodicity. We were also able to relate these novel aspects of physical activity to depression. Furthermore, this method allowed for discriminating noise patterns, which do not contribute to the complexity measures examined, from specific recurrent activity periods which do add to the calculated complexity measures.

Various resulting complexity measures significantly associated with a diagnosis of depression, whereas traditional measures such as mean level and diurnal rhythm measures did not. Moreover, complexity measures predicted the depression status better than the traditionally used actigraphy measures. This implies, first, that depressed people showed lower total duration of specific recurrent activities, such as walking, biking, or other sportive activities and less diversity in the durations of such activities. It may be these differences that mainly characterize how physical activity is different between depressed and non-depressed people. We should note, though, that in these actigraphy measures duration and diversity overlapped almost completely. Thus, people with longer duration of activities also showed higher diversity of activities. This means that in this study we are unable to differentiate between these two aspects of complexity. Second, the fact that the complexity measures performed better in discriminating the two groups than simply the mean value of physical activity is worth noting. Reduction in physical activity is known to be a defining characteristic of depression and studies using objective actigraphy have shown that depressed individuals have a lower level of physical activity than individuals without depression^12,53^. In this context, the lack of significant differences in physical activity between the depressed and non-depressed groups, which was also observed previously in a sub sample of the same study^54^, is striking. One possible reason could be that the MOOVD study which focused on mood and movement attracted individuals with higher activity to begin with. Further, large variations in the BDI-II scores between baseline and follow-up were observed in the depressed group (see Table 2). Hence the mean physical activity per person is possibly averaged over periods with differing levels of depressive symptoms, resulting in less significant differences between the two groups. Taken together, this study makes a case for using more complex measures to understand reduction in physical activity in depression and suggests the need for within subject studies to understand the same. A previous study suggested that more complex dynamic measures of variables of interest in the field of psychopathology would not be able to contribute more information than the mean^55^. This study, as well as other recent studies^56,57^ suggest otherwise. Third, as there was minimal overlap between the complexity and the traditional measures as shown by the correlations in Table 5, findings suggest that the complexity measures provide complementary information regarding activity patterns over and above currently used indicators. This finding is supplemented with a linear regression analysis (Supplementary 1), which showed that the goodness of fit obtained using recurrence-based variables are consistently higher than those achieved by classically used actigraphy variables.

This work was exploratory in nature, and should be considered as a first foray into studying the recurrence patterns of activity data in psychopathology. If the reported differences in complexity of physical activity patterns can be shown to contribute to the development of depressive symptoms, the RQA complexity measure may become a promising diagnostic tool. RQA has been recently proposed as a promising bio-marker to identify autism spectrum disorders from EEG data^58^. Similarly, RQA may be used to objectively evaluate the extent to which physical activity patterns of depressed individuals have a healthy level of complexity.

We have some recommendations for future research. First, it is relevant to explore whether changes in the complexity of physical activity patterns actually precede the onset of symptom changes in individuals with depression. RQA has been used to successfully predict sudden transitions in other fields^59,60^. The Transitions In Depression (TRANS-ID) study has collected unique personalized datasets in which people are followed intensively over the course of symptom transitions, including actigraphy measurements^61^. This is therefore the ideal design to test whether decreases in the complexity of physical activity precedes symptom transitions in depression. Another recommendation for future studies is to examine whether intervention on physical activity patterns in the direction of increased complexity in depressed patients would lead to a reduction in the level of symptoms.

A few limitations need to be considered when interpreting our study. One methodological limitation is that $13\%$ of the participants failed to show enough variation in actigraphy patterns to perform the RQA analyses. The latter analyses cannot be performed with the presence of too many zeros (perfect inactivity) in the data, as this would result in cluttering and consequent masking of information. Therefore, potential application in clinical practice should take into account that this method will not work for some patients who show too little activity. Another methodological issue is the sample size. Although, the power to calculate the complexity outcomes per person was high, as continuous actigraphy data were available for 4 weeks for each person leading to highly reliable values of complexity for every individual, the between-person power to compare group differences was lower. This may explain why none of the group comparisons regarding complexity outcomes were statistically significant after multiple testing corrections. Therefore, there is a need for replication of this finding with larger sample sizes.

## Conclusions

This study explored the association between physical activity and depression by studying the recurrent activity patterns that are present in the actigraphy data of depressed and non-depressed individuals. It is concluded that the diversity and average duration of activities was significantly associated with depression, while mean levels in physical activity and circadian rhythm variables were not. This novel finding has important implications for understanding how physical activity relates to mood disorders like depression. If future studies will replicate this finding and show support that complexity patterns causally relate to development of symptoms, RQA measures may constitute an additional tool for personalized diagnostics and treatment strategies, in depression.

## Supplementary Information

Supplementary Information.

## Data Availability

The datasets generated and/or analysed during the current study are not publicly available due to the nature of the data (intensive longitudinal actigraphy data), which cannot be considered fully anonymous. However, data are available from the corresponding author on reasonable request. The codes used for data analysis in this paper may be found at github.com/sgeorge91/ComplexityInDepression.

## References

[1] Dunn, A. L., Trivedi, M. H. & O’Neal, H. A. Physical activity dose-response effects on outcomes of depression and anxiety. In *Database of Abstracts of Reviews of Effects (DARE): Quality-assessed Reviews [Internet]* (Centre for Reviews and Dissemination (UK), 2001). 10.1097/00005768-200106001-00027 11427783

[2] Rebar AL (2015). A meta-meta-analysis of the effect of physical activity on depression and anxiety in non-clinical adult populations. Health psychology review.

[3] Teychenne M, Ball K, Salmon J (2010). Sedentary behavior and depression among adults: a review. International journal of behavioral medicine.

[4] Marques A (2020). Cross-sectional and prospective relationship between physical activity and depression symptoms. Scientific reports.

[5] Weyerer S, Kupfer B (1994). Physical exercise and psychological health. Sports Medicine.

[6] Farmer ME (1988). Physical activity and depressive symptoms: the NHANES I Epidemiologic Follow-up Study. American journal of epidemiology.

[7] Strawbridge WJ, Deleger S, Roberts RE, Kaplan GA (2002). Physical activity reduces the risk of subsequent depression for older adults. American journal of epidemiology.

[8] Teychenne M, Ball K, Salmon J (2008). Physical activity and likelihood of depression in adults: a review. Preventive medicine.

[9] Roshanaei-Moghaddam B, Katon WJ, Russo J (2009). The longitudinal effects of depression on physical activity. General hospital psychiatry.

[10] Rawson MJ (2000). Circadian and circaseptan components of blood pressure and heart rate during depression. Scr Med (Brno).

[11] Germain A, Kupfer DJ (2008). Circadian rhythm disturbances in depression. Human Psychopharmacology: Clinical and Experimental.

[12] Minaeva O (2020). Screening for depression in daily life: Development and external validation of a prediction model based on actigraphy and experience sampling method. J Med Internet Res.

[13] Levandovski, R. *et al.* Depression scores associate with chronotype and social jetlag in a rural population. *Chronobiol. Int.*10.3109/07420528.2011.602445 (2011). 10.3109/07420528.2011.602445 21895489

[14] Zumbach, G. & Lynch, P. Heterogeneous volatility cascade in financial markets. *Phys. A: Stat. Mech. Appl.* **298**, 521–529, 10.1016/S0378-4371(01)00249-7 (2001). arXiv:0105162.

[15] Vitale, J. A. *et al.* Chronotype influences activity circadian rhythm and sleep: Differences in sleep quality between weekdays and weekend. *Chronobiol Int.*10.3109/07420528.2014.986273 (2015). 10.3109/07420528.2014.986273 25469597

[16] Miller, M. A. *et al.* Chronotype predicts positive affect rhythms measured by ecological momentary assessment. *Chronobiol. Int.*10.3109/07420528.2014.983602 (2015). 10.3109/07420528.2014.983602 PMC4458846 25410882

[17] Burton, C. *et al.* Activity monitoring in patients with depression: asystematic review. *J. Affect. Disord.*10.1016/j.jad.2012.07.001 (2013). 10.1016/j.jad.2012.07.001 22868056

[18] Au, J. & Reece, J. The relationship between chronotype and depressive symptoms: ameta-analysis. *J. Affect. Disord.*. 10.1016/j.jad.2017.04.021 (2017). 10.1016/j.jad.2017.04.021 28463712

[19] Albert PS, Hunsberger S (2005). On analyzing circadian rhythms data using nonlinear mixed models with harmonic terms. Biometrics.

[20] Shinagawa M (2002). Seven-day (24-h) ambulatory blood pressure monitoring, self-reported depression and quality of life scores. Blood pressure monitoring.

[21] Maes, M., Meltzer, H. Y., Suy, E. & De Meyer, F. Seasonality in severity of depression: relationships to suicide and homicide occurrence. *Acta Psychiatrica Scand.*10.1111/j.1600-0447.1993.tb03431.x (1993). 10.1111/j.1600-0447.1993.tb03431.x 8249645

[22] Lang M, Krátkỳ J, Shaver JH, Jerotijević D, Xygalatas D (2015). Effects of anxiety on spontaneous ritualized behavior. Current Biology.

[23] Martín-Martínez D (2012). Nonlinear analysis of actigraphic signals for the assessment of the attention-deficit/hyperactivity disorder (adhd). Medical engineering & physics.

[24] Parro V, Valdo L (2018). Sleep-wake detection using recurrence quantification analysis. Chaos: An Interdisciplinary Journal of Nonlinear Science.

[25] Hu, K. *et al.* Non-random fluctuations and multi-scale dynamics regulation of human activity. *Phys. A: Stat. Mech. Appl.*10.1016/j.physa.2004.01.042 (2004). 10.1016/j.physa.2004.01.042 PMC2749944 15759365

[26] Ong, J. C., Arnedt, J. T. & Gehrman, P. R. Insomnia diagnosis, assessment, and evaluation. *Principles and Practi. Sleep Med.*10.1016/b978-0-323-24288-2.00083-0 (2017).

[27] Tazawa Y (2019). Actigraphy for evaluation of mood disorders: a systematic review and meta-analysis. Journal of affective disorders.

[28] Evenson, K. R., Catellier, D. J., Gill, K., Ondrak, K. S. & McMurray, R. G. Calibration of two objective measures of physical activity for children. *J. Sports Sci.*10.1080/02640410802334196 (2008). 10.1080/02640410802334196 18949660

[29] Kaplan, K. A., Talbot, L. S., Gruber, J. & Harvey, A. G. Evaluating sleep in bipolar disorder: comparison between actigraphy, polysomnography, and sleep diary. *Bipolar Disord.*10.1111/bdi.12021 (2012). 10.1111/bdi.12021 PMC3549461 23167935

[30] Booij SH (2015). Cortisol and *α*-amylase secretion patterns between and within depressed and non-depressed individuals. PloS one.

[31] Stavrakakis N (2015). Temporal dynamics of physical activity and affect in depressed and nondepressed individuals. Health Psychology.

[32] Beck AT, Steer RA, Brown GK (1996). Beck depression inventory-II.. San Antonio.

[33] Heil, D. P. Predicting activity energy expenditure using the actical® activity monitor. *Res. Quart. Exerc. Sport*10.1080/02701367.2006.10599333 (2006). 10.1080/02701367.2006.10599333 16646354

[34] Pompe, B. Ranking and entropy estimation in nonlinear time series analysis. *Nonlinear Anal. Physiol. Data*10.1007/978-3-642-71949-3_5 (1998).

[35] Conover, W. J. & Iman, R. L. The rank transformation as a method of discrimination with some examples. *Commun. Stat. Theory Methods*10.1080/03610928008827895 (1980).

[36] Ziegel, E., Press, W., Flannery, B., Teukolsky, S. & Vetterling, W. Numerical recipes: the art of scientific computing. *Technometrics*10.2307/1269484 (1987).

[37] Marwan N, Webber CL, Macau EE, Viana RL (2018). Introduction to focus issue: Recurrence quantification analysis for understanding complex systems. Chaos: An Interdisciplinary Journal of Nonlinear Science.

[38] Jenkins BN, Hunter JF, Richardson MJ, Conner TS, Pressman SD (2020). Affect variability and predictability: Using recurrence quantification analysis to better understand how the dynamics of affect relate to health. Emotion.

[39] Lichtwarck-Aschoff, A., Hasselman, F., Cox, R., Pepler, D. & Granic, I. *A characteristic destabilization profile in parent-child interactions associated with treatment efficacy for aggressive children* (Psychology, and Life Sciences, Nonlinear Dynamics, 2012). 22695153

[40] Marwan N, Romano MC, Thiel M, Kurths J (2007). Recurrence plots for the analysis of complex systems. Physics reports.

[41] Thiel M, Romano MC, Read P, Kurths J (2004). Estimation of dynamical invariants without embedding by recurrence plots. Chaos: An Interdisciplinary Journal of Nonlinear Science.

[42] Eroglu, D., Marwan, N., Prasad, S. & Kurths, J. Finding recurrence networks’ threshold adaptively for a specific time series. *Nonlinear Proces. Geophys.*10.5194/npg-21-1085-2014 (2014).

[43] Afsar O, Tirnakli U, Marwan N (2018). Recurrence quantification analysis at work: quasi-periodicity based interpretation of gait force profiles for patients with parkinson disease. Scientific reports.

[44] Javorka, M. *et al.* Recurrences in heart rate dynamics are changed in patients with diabetes mellitus. *Clin. Physiol. Functi. Imaging*10.1111/j.1475-097X.2008.00813.x (2008). 10.1111/j.1475-097X.2008.00813.x 18507669

[45] Rusinek R, Zaleski K (2016). Dynamics of thin-walled element milling expressed by recurrence analysis. Meccanica.

[46] Rusinek, R., Lajmert, P., Krzysztof, K., Kruszynski, B. & Warminski, J. Chatter identification methods on the basis of time series measured during titanium superalloy milling. *Int. J. Mech. Sci.*10.1016/j.ijmecsci.2015.05.013 (2015).

[47] Witting W, Kwa I, Eikelenboom P, Mirmiran M, Swaab DF (1990). Alterations in the circadian rest-activity rhythm in aging and alzheimer’s disease. Biological psychiatry.

[48] Van Someren EJ (1999). Bright light therapy: improved sensitivity to its effects on rest-activity rhythms in alzheimer patients by application of nonparametric methods. Chronobiology international.

[49] Kunkels, Y. K. *et al.* ACTman: automated preprocessing and analysis of actigraphy data. *J. Sci. Med. Sport*10.1016/j.jsams.2019.11.009 (2019). 10.1016/j.jsams.2019.11.009 31813761

[50] Welch BL (1947). The generalization ofstudent’s’ problem when several different population variances are involved. Biometrika.

[51] Cohen J (2013). Statistical power analysis for the behavioral sciences.

[52] Virtanen P (2020). Scipy 1.0: fundamental algorithms for scientific computing in python. Nature methods.

[53] Difrancesco, S. *et al.* Sleep, circadian rhythm, and physical activity patterns in depressive and anxiety disorders: a 2-week ambulatory assessment study. *Depression Anxi.* (2019). 10.1002/da.22949 PMC6790673 31348850

[54] Stavrakakis, N. *et al.* The dynamic relationship between physical activity and mood in the daily life of depressed and non-depressed individuals: a within-subject time-series analysis. *Phys. Activ. Depres. Symptoms* **129**, (2015).

[55] Dejonckheere, E. *et al.* Complex affect dynamics add limited information to the prediction of psychological well-being. *Nat. Hum. Behav.*10.1038/s41562-019-0555-0 (2019). 10.1038/s41562-019-0555-0 30988484

[56] Kuranova, A. *et al.* Measuring resilience prospectively as the speed of affect recovery in daily life: acomplex systems perspective on mental health. *BMC Med.*10.1186/s12916-020-1500-9 (2020). 10.1186/s12916-020-1500-9 PMC7027206 32066437

[57] Schreuder, M. J. *et al.* Early warning signals in psychopathology: what do they tell?. *BMC Med.*10.1186/s12916-020-01742-3 (2020). 10.1186/s12916-020-01742-3 PMC7557008 33050891

[58] Heunis, T. *et al.* Recurrence quantification analysis of resting state EEG signals in autism spectrum disorder—a systematic methodological exploration of technical and demographic confounders in the search for biomarkers. *BMC Med.*10.1186/s12916-018-1086-7 (2018). 10.1186/s12916-018-1086-7 PMC6027554 29961422

[59] George, S. V., Kachhara, S., Misra, R. & Ambika, G. Early warning signals indicate a critical transition in betelgeuse. *Astron. Astrophys.* (2020).

[60] Kabiraj L, Saurabh A, Nawroth H, Paschereit CO (2015). Recurrence analysis of combustion noise. AIAA Journal.

[61] Helmich, M. A. *et al.* Transitions in depression (trans-id) recovery: Study protocol for a repeated intensive longitudinal $$n= 1$$ study design to search for personalized early warning signals of critical transitions towards improvement in depression. *PsyArXiv* (2020).

