# Supplementary material for: Uncovering complexity details in actigraphy patterns to differentiate the depressed from the non-depressed
Source: Sci Rep. 2021 Jun 29;11:13447. doi: 10.1038/s41598-021-92890-w (PMC8241993; doi:10.1038/s41598-021-92890-w)
Supplement: Supplementary file 1 — Supplementary Information. [file 41598_2021_92890_MOESM1_ESM.pdf]

Supplementary 1: Logistic regression analysis

In this supplementary we present the results of the logistic regression analysis to predict depression status using the classical approaches considered in the paper and the novel recurrence plot variables. Initially we consider the best performing recurrence plot variable from our t-test analysis, namely the LAM/DET and the best performing classical actigraphy variable, namely the mean physical activity. The model is defined as follows.

$$Status \sim BestVar$$

Where the *BestVar* is either LAM/DET or mean physical activity. The results are listed under model 1 in Table S1.

Next, we consider all possible classical actigraphy variables and all possible recurrence plot variables. The model is defined as

$$Status \sim AllVar$$

Where *AllVar* includes LAM/DET, DET, LAM, Lavg, Lent, Vavg and Vent for recurrence plot based quantifiers and mean physical activity, IV, IS and RA for classical quantifiers. The results are listed under model 2 in Table S1.

Finally, we explore whether using a combination of classical and recurrence plot variables predicts depression status better than each individually. In this case we define the model as

$$Status \sim EveryVar$$

Where *EveryVar* includes all possible recurrence plot and classical variables. The results are listed under model 3 in Table S1.

Two measures are popularly used to report pseudo R^2 values, the McFadden R^2 and the normalized Cox-Snell R^2 (also called the Nagelkerle R^2)(Cox & Snell, 1990; Nagelkerke,

1991; Scott et al., 1991). Since there exists no consensus on which represents the goodness of fit better, we report both values in Table 2. While the values themselves change depending on the type of pseudo R^2 used, recurrence based variables outperform classical variables in both cases. Further, we see that using both variables together have a higher predictive value than using each individually, suggesting that the two quantifiers give complementary information. All analyses was conducted in R v 3.6.1 with the pseudo R^2 values being calculated using the "DescTools" library(Signorell, 2017).

Table S1: The goodness of fit defined using the Mcfadden and Nagelkerle pseudo R^2 indices for the three models defined above.

	**Classical variables**		**Recurrence based variables**	
	**McFadden R^2**	**Nagelkerle R^2**	**McFadden R^2**	**Nagelkerle R^2**
**Model 1**	0.033	0.060	0.089	0.154
**Model 2**	0.137	0.230	0.194	0.313
**Model 3**			0.275	0.422

Cox, D. R., & Snell, E. J. (1990). Analysis of Binary Data. *Biometrics*. https://doi.org/10.2307/2531476

Nagelkerke, N. J. D. (1991). A note on a general definition of the coefficient of determination. In *Biometrika*. https://doi.org/10.1093/biomet/78.3.691

Scott, A. J., Hosmer, D. W., & Lemeshow, S. (1991). Applied Logistic Regression. *Biometrics*. https://doi.org/10.2307/2532419

Signorell, A. (2017). DescTools: Tools for descriptive statistics. R package version 0.99.20. *CRAN*. https://doi.org/10.1016/j.foreco.2014.09.033
